# Evaluating the Psychometric Properties of Arabic Version of the Patient Health Questionnaire-9 (PHQ-9) among Omani Patients with End Stage Renal Disease on Dialysis

**DOI:** 10.1192/j.eurpsy.2025.1220

**Published:** 2025-08-26

**Authors:** O. S. Alkalbani, M. Al Alawi, S. Al Farsi

**Affiliations:** 1 psychiatry, omsb; 2 psychiatry, squh, muscat, Oman

## Abstract

**Introduction:**

Depression significantly affects patients with chronic kidney disease (CKD), with prevalence rates reaching up to 39% among those on hemodialysis, and is often overlooked in screening. This study aims to assess the PHQ-9’s effectiveness in Omani dialysis patients, potentially improving early detection and mental health care integration.

**Objectives:**

The objectives of this study are as follows: 1) To assess the sensitivity, specificity, positive predictive value and negative predictive value of the PHQ-9 in detecting depression among Omani renal dialysis patients at Al Seeb and Bausher dialysis units from October 2023 to January 2024. 2) To evaluate the psychometric properties of the PHQ-9, including the optimal cut-off score, internal consistency, and criterion validity, in Omani renal dialysis patients by January 2024

**Methods:**

This cross-sectional study was conducted from October 1, 2023, to January 31, 2024, at two renal dialysis centers in Muscat, focusing on adults aged 18 and older who had undergone dialysis for at least three months. Data collection included a sociodemographic questionnaire, the Patient Health Questionnaire (PHQ-9) for depression symptoms, and the Structured Clinical Interview for DSM-5 (SCID-5) for psychiatric evaluation, with all ethical standards adhered to and Institutional Review Board approval obtained. Data analysis utilized MedCalc® software, with statistical significance set at p < 0.05, and the diagnostic accuracy of the PHQ-9 evaluated through ROC curve analysis.

**Results:**

The study included 209 patients with Chronic Kidney Disease (CKD), averaging 48.43 years, with demographics summarized in **Table 1**. The Patient Health Questionnaire-9 (PHQ-9) effectively screened for Major Depressive Disorder (MDD), achieving an AUC of 0.87, as illustrated in **Figure 1**, with an optimal cutoff score of 9, sensitivity of nearly 78%, and specificity of about 85%. Additional metrics are detailed in **Table 2**, confirming the PHQ-9’s overall accuracy of 83.25% in identifying depression, highlighting the importance of clinical evaluation for diagnosis.

**Image 1:**

**Figure:**
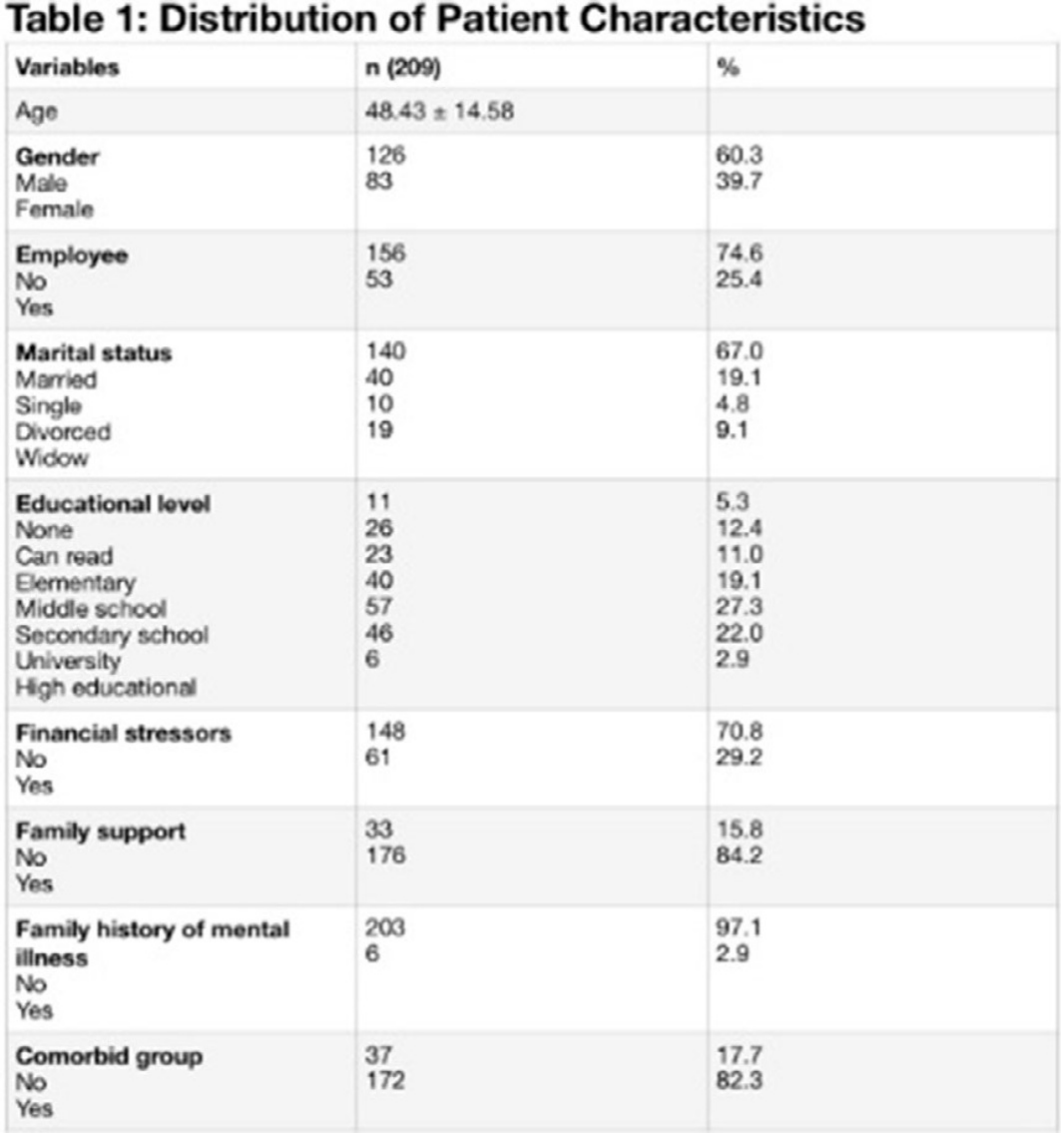

**Image 2:**

**Figure:**
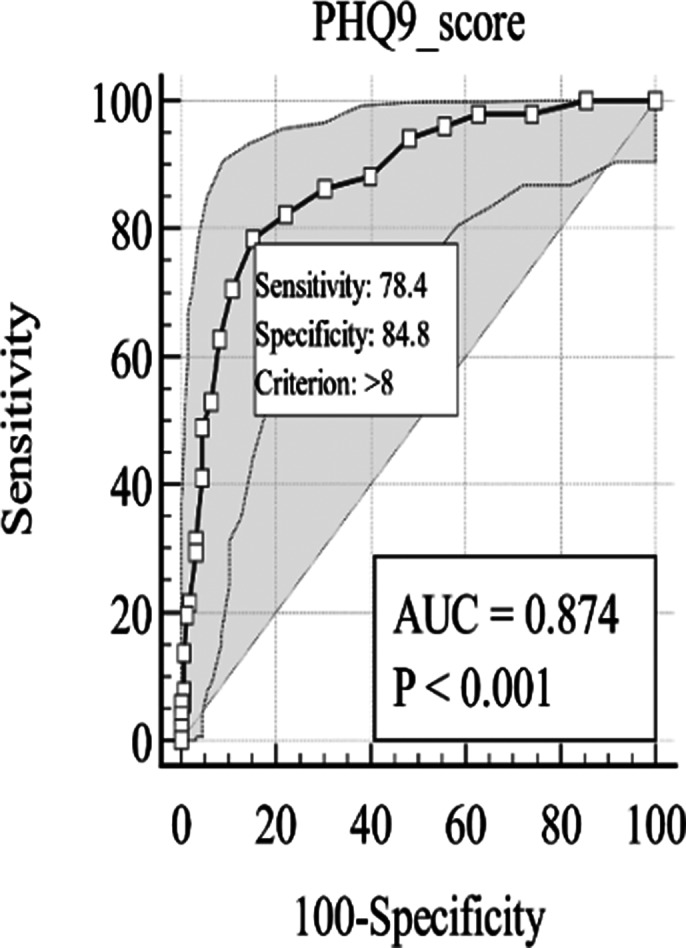

**Image 3:**

**Figure:**
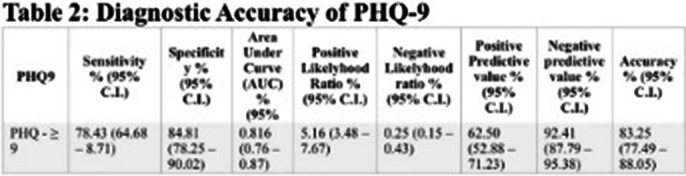

**Conclusions:**

This study provides strong evidence for the validity and reliability of the PHQ-9 as a depression screening tool for Omani dialysis patients, emphasizing the need for culturally sensitive approaches in screening. It suggests adjusting cutoff scores to enhance diagnostic accuracy, with significant implications for clinical practice in Oman and similar regions. Healthcare professionals are urged to consider cultural factors when implementing screening protocols to improve patient care. Future research should broaden the validation of the PHQ-9 across other regions in Oman and the Middle East, examining how linguistic and educational differences may affect its effectiveness.

**Disclosure of Interest:**

None Declared

